# Heterogeneity in the formation of primary and secondary visual fields during human prenatal development

**DOI:** 10.1186/s40659-024-00576-0

**Published:** 2024-11-28

**Authors:** Olga Godovalova, Alexandra Proshchina, Anastasia Kharlamova, Valeriy Barabanov, Yuliya Krivova, Olga Junemann, Marina Shahina, Sergey Saveliev

**Affiliations:** 1 Avsyn Research Institute of Human Morphology of Federal State Budgetary Scientific Institution “Petrovsky National Research Centre of Surgery”, Tsurupi Street, 3, Moscow, 117418 Russia; 2 The National Research Council Institute CNR-NANOTEC, Piazzale Aldo Moro 5, 00185 Rome, Italy; 3 Krasnopolsky Moscow Regional Research Institute of Obstetrics and Gynecology, Pokrovka Street, 22A, Moscow, 101000 Russia

**Keywords:** Human fetal development, Calcarine sulcus, Parietooccipital sulcus, Visual fields, Human fetuses, Occipital lobe, Gyrification

## Abstract

The human neocortex has a huge surface area with unique cytoarchitectonics, most of which is concealed in sulci. Some cytoarchitectonic fields are associated with macroscopic landmarks. In particular, the primary visual field 17 is associated with the calcarine sulcus. During the prenatal development of the human brain, neocortical gyri and sulci undergo changes and modifications after primary formation. To explore the morphogenetic processes in visual fields during the formation of the primary (provisional) and secondary (permanent) sulci, the occipital lobe of the human fetal brain was studied using immunohistochemical methods. The distribution of various glial and neuronal markers (S-100, β-III-tubulin, NeuN, reelin) in the calcarine sulcus and parietooccipital sulcus was compared. The heterogeneity in the formation of primary and secondary visual fields was demonstrated. The study revealed that the development of the primary visual field 17, linked with the calcarine sulcus, preceded the development of a shared anlage of fields 18 and 19 linked with the parietooccipital sulcus. The functional differentiation of the primary visual field begins during the period of thalamic afferent ingrowth. This process coincides with the temporal smoothing of the calcarine sulcus, indicating a simultaneous progression of functional specialization and structural modifications. At the late fetal period, cortical plate of gyri and sulci banks showed higher NeuN-labeling than inside the sulcus in the same cytoarchitectonic field.

**Supplementary Information:**

The online version contains supplementary material available at 10.1186/s40659-024-00576-0.

## Introduction

Forebrain formation and maturation are prominent topics in recent neurobiology given that the forebrain cortex serves as the functional foundation for human cognition and intelligence. Extensive research on cortex formation has yielded substantial knowledge. The formation, migration, and differentiation of neuro- and glioblasts in the cortex have been studied on various mammals, particularly rats [1, 2], and primates [3–5]. However, it is important to note that findings from the model experiments cannot be directly extrapolated to human brain development [6–8]. The human brain possesses molecular-genetic and morphological features that cannot be studied in cell cultures or using model animals. Notably, rodents’ brains are lissencephalic making it impossible to study the formation of sulci and gyri in these model subjects. This apparent limitation is a significant challenge in global neurobiology [8].

The human neocortex has a vast surface area with unique cytoarchitectonics, most of which is concealed in sulci. It is known that the position and extent of the cytoarchitectonic fields of the human visual cortex have characteristic relations to the occipital sulci, their banks and fundi, and to the points of intersection of the sulci [9]. A spatial correlation between the primary visual area (17) and the calcarine sulcus (*sulcus calcarinus*, Cas) has been demonstrated [10–12]. The spatial correlation between occipital sulci and visual areas defined by functional neuroimaging techniques in humans has also been established for multiple retinotopic areas [13–17] However, knowledge of how cytoarchitectonic fields are formed during human brain development is still fragmented. The ingrowth of fibers from the retina to lateral geniculate nucleus, the ingrowth of geniculocortical axons to the visual cortex and the role of the subplate zone in the development of the visual cortex have been studied in monkeys and humans [18–23], and neurochemical compartmentation of different cortical layers of the primary visual field has been demonstrated [24–27]. However, the maturation process within the sulci of the occipital lobe remains unexplored.

During human prenatal brain development, temporary (provisional, primary) sulci are formed [28–30]. These primary sulci represent temporary grooves that appear and subsequently disappear, replaced by secondary (permanent) sulci. We have previously demonstrated that the primary calcarine sulcus and parietooccipital sulcus (*sulcus parietooccipitalis*, Pos) emerge as early as the 12th gestational week (gw). The Pos temporarily smoothes out at 18–20 gw. The Cas smoothes out later in development, at 23–25 gw [30], and is regularly detected on ultrasound only after 24–25 gw [31, 32]. Whether earlier smoothing of the Pos may influence the development and maturation of the cortical plate in this area remains unclear.

In this study, we aimed to describe the patterns of maturation of the primary and secondary cytoarchitectonic visual fields, considering the complex morphogenesis of provisional and permanent sulci. As a result, new data with insights into the dynamics of distribution of certain key neurodevelopmental markers: astrocytic differentiation (S-100), neural differentiation (β-III-tubulin), neuron maturation marker (NeuN) and neuronal migration marker (Reelin) were obtained.

Neuronal nuclear protein (NeuN) is a well-recognized marker that is detected exclusively in post-mitotic mature neurons in nearly all parts of the vertebrate nervous system [33]. Reelin is a signaling molecule produced by the Cajal-Retzius cells at the marginal zone. Reelin is released into the surrounding matrix and serves as both an attractant and stop-signal involving cadherin and nectin molecules for the migrating neurons [34, 35]. Calcium-binding protein S-100 (glial marker in the mature brain) may act as a growth-differentiating factor for neurons and glia [36] and as a regulator of synaptic plasticity [37]. β-III-tubulin is cytoskeletal protein, one of the earliest markers of neural tissue, expressed in both the central and peripheral nervous system [38–40].

## Materials and methods

### Tissue sources and processing

Postmortem fetal brain tissues from the Collection of the Laboratory of Nervous System Development of Avtsyn Research Institute of Human Morphology, Moscow were examined [41]. All the material was taken and handled in accordance with Russian legislation and international standards and regulations, including the Declaration of Helsinki. The study received approval from the local Ethics Committee of Avtsyn Research Institute of Human Morphology. A total of 56 brain (112 hemispheres) autopsies from fetuses aged from twelve weeks of gestation to birth were analyzed in this study. Several factors were considered during the analysis of the specimens, including sex, gestational age, clinical diagnosis of both the mother and the fetus, reasons for pregnancy termination, and causes of fetal death. For macromorphological and histological analysis, samples with no clinical data on chromosomal abnormalities and CNS pathologies were selected. Samples for immunohistochemistry were selected after histological evaluation of the preservation of the material. Samples with focuses of hemorrhage in the neocortex, necrosis, or signs of postmortem autolysis were excluded. Samples with prolonged storage in formalin were also excluded since this may influence the distribution of the immunoreactivity. Gestational age was estimated from clinical history as the time the last menstrual period and was determined by ultrasonography and then specified by weight/height and crown-rump length [42]. The presence of the Cas and Pos was visually noted in the preparations and the data was recorded in a table. Macrophotos of the medial surface of the hemispheres were taken (camera CASIO QV5700).

### Histological preparation and immunohistochemistry

Immunohistochemical examination was conducted for the brains of 11 human fetuses at various stages of development, 14 premature newborns, and 2 full-term newborns. The features of the specimens and estimated ages are outlined in Supplement, Table 1. Depending on the age of the fetuses, either the entire brain or a section of the hemispheric medial wall containing Cas and Pos was examined. The specimens were fixed by immersion in 10% acidic formalin, or buffered formalin (pH 7.0–7.4), or Bouin’s solution. Subsequently, the specimens were dehydrated in alcohols of increasing concentration and dioxane, embedded in paraffin, and cut into a series of 10-µm-thick sections for each sample. These sections were stained with classical histological Cresyl violet (Nissl) staining to provide a cytoarchitectonic overview.

Additionally, immunohistochemical analysis was conducted. Primary antibodies targeting specific antigens of the nervous system: neuronal nucleus antigen (NeuN), neuron-specific β-III tubulin, Ca2+-binding neuroglial protein (S-100), and reelin were used (Table 1). The sections were glued onto Superfrost slides (Thermo Fisher Scientific Inc., Fremont, CA, USA) and stored at 4 °C.

Negative control sections, in which primary antibodies were replaced with “Dako diluent” (Dako) or PBS with a pH of 7.2–7.4 (0.01 M), were included in each immunostaining procedure for each case, and non-specific were absent in all negative controls.

Positive controls involved reactions conducted on late fetal brain tissue samples.

**Table 1:** Details of immunohistochemical techniques

Primary antibodies	Antigen retrieval	Dilution	Time and incubation conditions	Visualization kit	Chromogen
Rabbit antibodies to S-100 (Sigma-Aldrich Cat# S2644, Darmstadt, Germany, RRID: AB_477501)	No	1:300	18–20 h at 8 C˚	HRP-F(ab’)2α rabbit IgG, 1:500 (Thermo Fisher Scientific Inc., Kalamazoo, MI, USA Cat# A24531, RRID: AB_2535999)	DAB*
Rabbit polyclonal antibodies to neuron-specific β-III tubulin (Abcam Cat# ab229590, Cambridge, CB2 0AX, UK, RRID: AB_2827733)	No	1:1000 1:2000	1 h at room t0	Ultra Vision LP Detection System (Thermo Fisher Scientific Inc., Kalamazoo, MI, USA)	DAB
Mouse monoclonal antibody to NeuN clone A60 (Millipore Cat# MAB377, Darmstadt, Germany, RRID: AB_2298772)	Yes	1:100	1 h at room t0	Ultra Vision LP Detection System (Thermo Fisher Scientific Inc., Kalamazoo, MI, USA)	DAB
Mouse monoclonal antibody to reelin clone 142 (Millipore Cat# MAB5366, Darmstadt, Germany, RRID: AB_2285132)	No	1:250	1 h at room t0	Ultra Vision Plus Detection System (Thermo Fisher Scientific Inc., Kalamazoo, MI, USA)	DAB

*DAB solution – 0.05% solution of DAB (3–3’-diaminobenzidine, Sigma-Aldrich) in 0.05 M Tris-HCl – buffer solution, pH 7.6, containing 0.02% H_2_O_2_

### Microscopy, imaging, and morphometric analysis

All preparations were analyzed using a light microscope (DM 2500; Leica Microsystems, Wetzlar, Germany) equipped with a digital camera (Lomo, Saint Petersburg, Russia) and the McrA-View 7.1.1.2 software (Lomo, Saint Petersburg, Russia). Measurements were performed on the microphotographs of preparations. Some preparations were scanned using a modified MEKOS-C2 complex (MEKOS, Russia) based on a Zeiss Axio Imager 1 microscope (Carl Zeiss QEC GmbH, Germany) with an x20 lens magnification.

For each sample, for both Cas and Pos, the immunoreactive area coefficient was measured in at least 12 non-overlapping observation fields at magnification x400 (for reelin labeling) or x100 (for S-100, NeuN labeling). To exclude interlayer differences, for S-100 and NeuN markers, we selected observation fields that included all layers (II-VI) of the cortical plate. To select subplate micrographs, we oriented the Nissl sections to cover the entire thickness of the zone. 12 subplate non-overlapping observation fields were photographed under Pos and Cas. The size of the fields depended on the thickness of the zone, but it was similar within the same age. To count the reelin-immunoreactive area coefficient, 12 non-overlapping observation fields of the marginal zone in each sulcus were examined. The extracellular matrix was taken into account; Cajal-Retzius cells were excluded from counting. Photographs were taken from several sections for each marker.

To evaluate the immunoreactive patterns in the Cas and Pos regions, the coefficient of the immunoreactive area was calculated using the Image J software (version 1.43, Ferreira, T. & Rasband, W.) for reelin, NeuN, and S-100 labeling. In microphotographs with the areas of interest, color pixels were highlighted using color threshold tool adjusted to a fixed parameters [43] Then coefficient of immunoreactivity (the ratio of the sum of immunopositive (color) pixels to the total number of pixels in the selected area was calculated using Image J algorithms.

### Statistical analysis

To evaluate the significance of differences in the presence or absence of Cas and Pos at different developmental intervals, the Fisher’s test was applied. Fisher’s criterion was calculated separately for each sulcus depending on gestational age. The *p*-value < 0.05 was considered statistically significant.

For the statistical analysis of the immunoreactivity coefficient, the fetal period was classified into three stages (early fetal (13–20 weeks), middle fetal (21–28 weeks), and late fetal (29–40 weeks) periods [44]. Additionally, the ratio of the immunoreactivity coefficient in Cas and Pos was compared at the stages of smoothing of primary sulci and the appearance of permanent sulci instead of them.

Statistical analysis was performed in Statistica 10 (Statsoft Inc., Tulsa, USA) using the nonparametric Kruskal-Wallis ANOVA with post-hoc multiple comparisons of mean ranks of all pairs. To compare the immunoreactivity between the Cas and Pos sulci, the Wilcoxon matched pairs test with Bonferroni correction was used.

## Results

### Macrocharacteristics of the development of the calcarine and parietooccipital sulci

Firstly, introduce the definitions used in this study. The primary sulcus is a visible temporary cavity in the surface of the cerebral hemispheres at the early stages of their formation. Further, the primary sulcus smooths out, and a permanent sulcus with the same topographic characteristics soon forms in its place. Sulcus smoothing – is a visible absence of sulcus on its greater length at macromorphological level in comparison with the previous studied stages. According to our observations, the middle part of the calcarine sulcus is smoothed most often. A few millimeters of the anterior and posterior parts of the sulcus may be preserved, as we seen in some studied cases (Supplemental Fig. 2c, d).

**Fig. 1:**
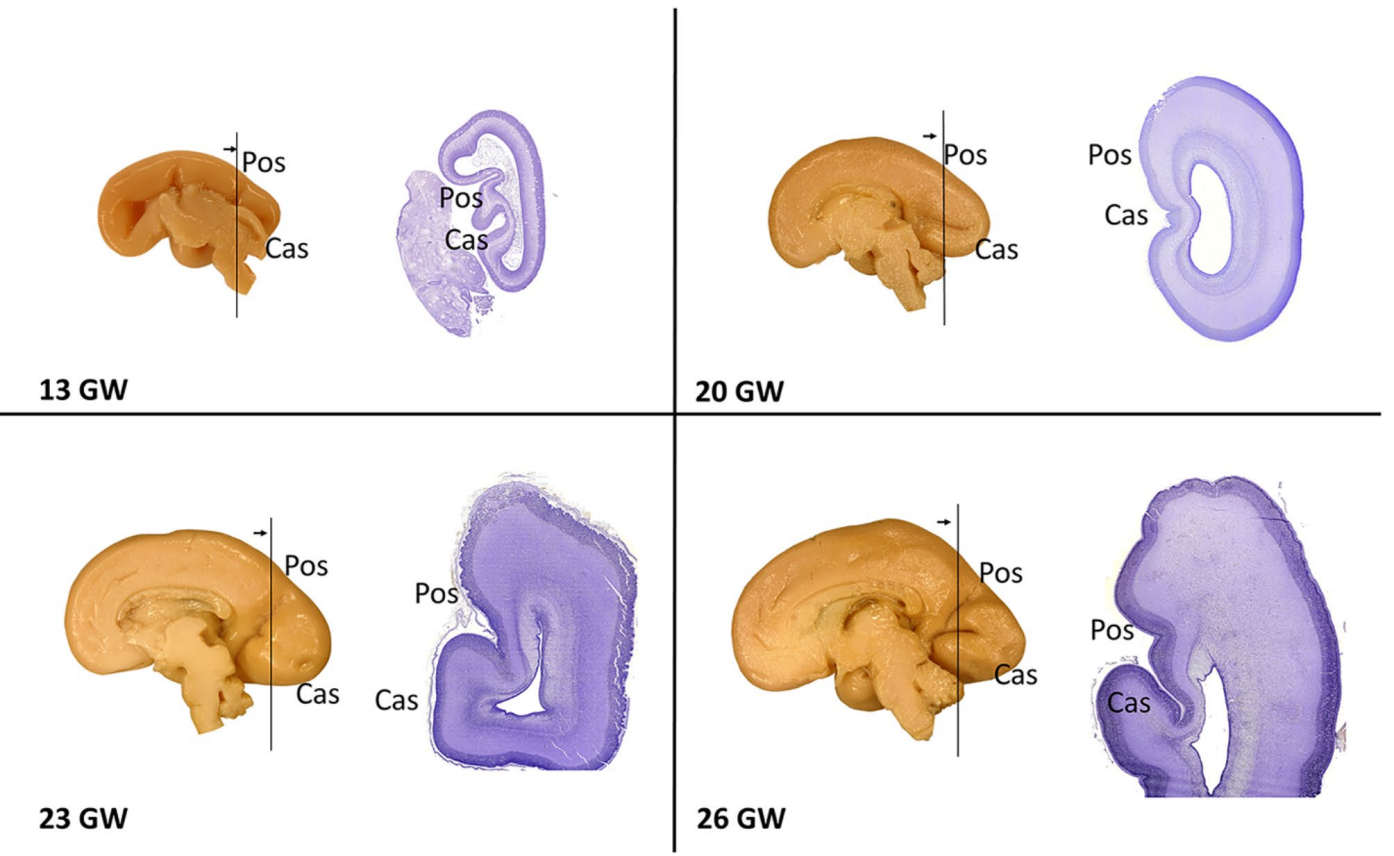
Stages of the development of the calcarine (Cas) and parietooccipital sulci (Pos) during the human prenatal ontogenesis. Medial surface of the hemispheres of samples 13, 20, 23 and 26 gw, on the right are Nissl-stained histological coronal sections, right hemispheres

According to our data at the macromorphological level, the Cas and Pos primary sulci became clearly visible as early as 12 gw (Table 2). These primary sulci appeared as folds in the entire medial hemispheric wall. This arrangement of sulci on the medial brain surface was observed until 17 gw (Fig. 1). At 17–20 gw (5th lunar month), a temporary smoothing of the Pos was seen. At these stages, the Pos was not visible in 4 cases of 11 on both hemispheres (*p* = 0.000049, Table 2; Fig. 1). After the 21st gw, the Pos was present in all cases. From 21 to 24 gw (6th lunar month), the smoothing of the Cas was a significant event (*p* = 0.000145, Table 2; Fig. 1), typically occurring at 22–24 gw. Cas was smoothed in 4 cases on both hemispheres, and in two cases Cas was detected in only one of the hemispheres. From 25 to 40 gw (7th–10th lunar months), the Cas was found in almost all preparations except for one hemisphere of the 25 gw fetus. The significance of differences was evaluated using Fisher’s test.

In general, frequent Pos smoothing was observed at 17–20 gw, and frequent full or partial Cas smoothing was observed at 21–25 gw.

**Table 2:** Formation of calcarine and parietooccipital sulci depending on age. Percentages are given as a proportion of the total number of specimens in each age group

Age, gw	13–16	17–20	21–24	25–28	29–32	33–36	37–40
Calcarine sulcus	8 (100%)	10.5 (96%)	6 (55%)	7.5^2^ (94%)	6 (100%)	5 (100%)	4 (100%)
Parieto-occipitale sulcus	8 (100%)	7 (64%)	11 (100)	8 (100%)	6 (100%)	5 (100%)	4 (100%)
N^1^	8	11	11	8	6	5	4

^1^ N – the number of fetuses per period

^2^ In the case of the presence of sulci on two hemispheres of the brain, we assigned 1 point to the specimen. In the case a sulcus was presented only in one hemisphere, the result was evaluated as 0.5 points

### Immunohistochemical analysis of neuronal nuclear histone protein NeuN

At 12–13 gw, the primary folds of the entire hemispheric wall, Cas and Pos, were already visible on the medial surface of the occipital lobe. It was possible to identify a typical structure organized as follows: the lower part of the hemispheric wall bordering the ventricular cavity formed by a broad dense cellular ventricular zone (*zona ventricularis*, VZ); above it was the subventricular zone (*zona subventricularis*, SVZ), then the narrow intermediate zone (*zona intermedia*, IZ), in which radial glia fibers and neuroblasts migrating along them towards the cortical plate (*lamina corticalis*, CP) were clearly visible; the subplate (*zona sublaminaris*, SP) border was not yet clearly visualized, and the closest to the surface of the pia mater was the marginal zone (*zona marginalis*, MZ). The thickness of the CP inside the sulci and in straight parts of the CP did not differ visually.

**Fig. 2:**
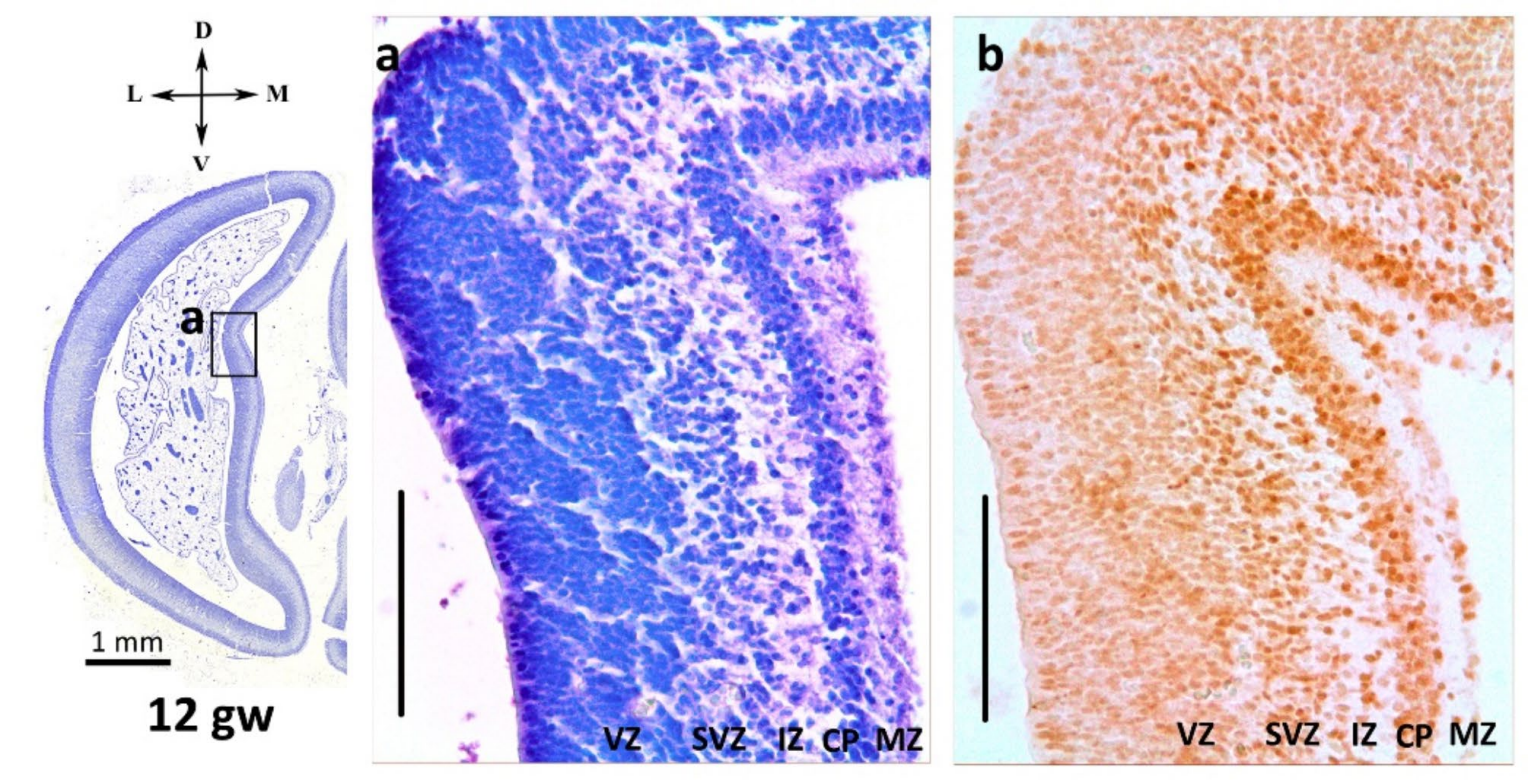
Parietooccipital sulcus (Pos) at 12th gw. The forming cortical plate was clearly visible on Nissl staining (**a**) and in NeuN-immunohistochemical labeling (**b**). NeuN-immunopositive neuroblasts were already seen in the subventricular (SVZ), intermediate zone (IZ), cortical plate (CP) and marginal zone (MZ). Right hemisphere. Coronal section. Scale bar: 100 µm

NeuN-immunopositive neuroblasts were already detected in SVZ, IZ, CP, and MZ, and in the CP, such neuroblasts were more frequent (Fig. 2). The distribution of NeuN labeling did not differ between sulci.

**Fig. 3:**
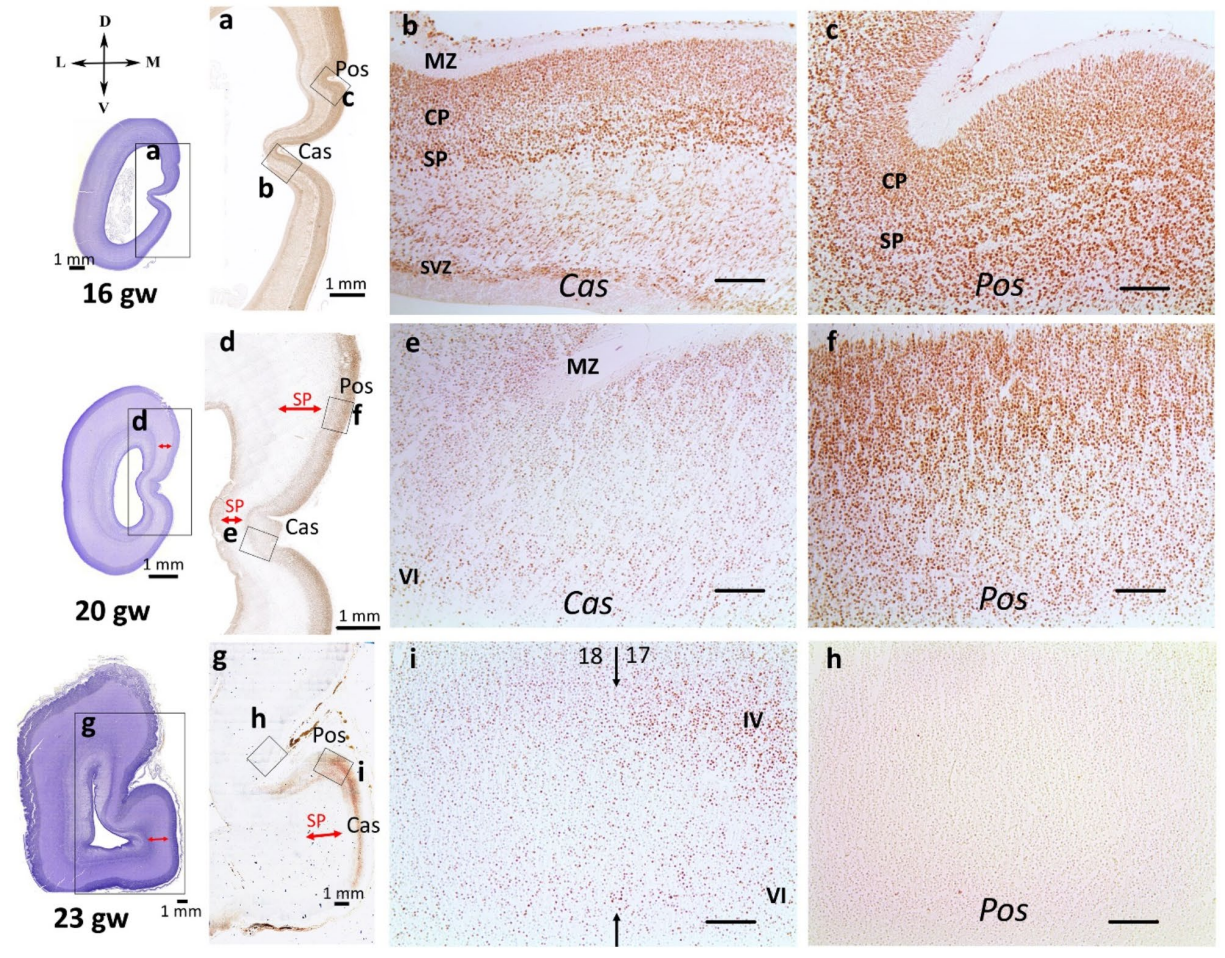
NeuN-immunohistochemical staining, coronal sections, occipital lobe at 16 gw, right hemisphere (**a**, **b**, **c**), 20 gw, right hemisphere (**d**, **e**, **f**), 23 gw, right hemisphere (**g**, **I**, **h**). The red arrow indicates the subplate zone. SVZ – subventricular zone, IZ – intermediate zone, SP – subplate zone, CP – cortical plate, MZ – marginal zone. Scale bar: 100 μm

**Fig. 4:**
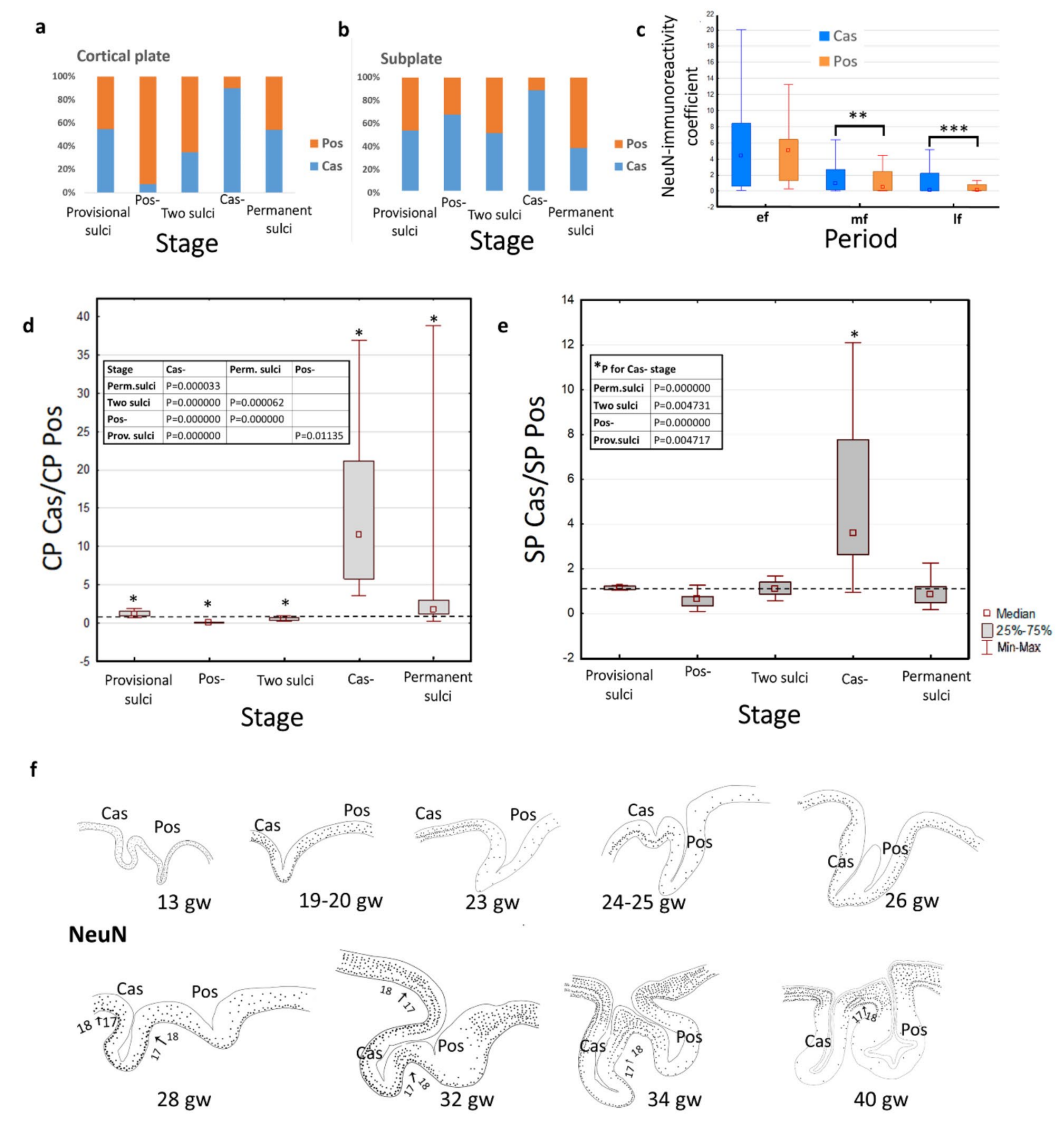
(**a**) – Percentage ratio of NeuN-immunoreactivity coefficient in the cortical plate of calcarine sulcus and parietooccipital sulcus. (**b**) – Percentage ratio of NeuN-immunoreactivity coefficient in the subplate under calcarine sulcus and parietooccipital sulcus. (**с**) – NeuN-immunoreactivity coefficient in early fetal (ef), middle fetal (mf) and late fetal (lf) periods. Significant differences between sulci are marked by square brackets and asterisks (**p = 0.005036, ***p = 0.000001). (**d**) – Boxplots and multiple comparison for ratio of the NeuN-immunoreactivity coefficient in the cortical plate of calcarine sulcus and parietooccipital sulcus. (**e**) – Boxplots and multiple comparison for ratio of the NeuN-immunoreactivity coefficient in the subplate of calcarine sulcus and parietooccipital sulcus. Comparable stages: provisional sulci; the parietooccipital sulcus smoothing stage (Pos-); two sulci, when both sulci exist; the calcarine sulcus smoothing stage (Cas-). Significant differences between stages are marked by square brackets and asterisks or presented in the table to the right of the bosxplots. (**f**) –Scheme of the distribution of NeuN-immunoreactive cells in the cortical plate at different stages of fetal development, Cas - calcarine sulcus, Pos - parietooccipital sulcus

At 14–18 gw, Cas and Pos were well defined. At these stages of development, Cas and Pos were folds of the entire hemispheric wall. In sections stained with NeuN, the differences in the thickness of SP were clearly visible. NeuN-immunoreactive cells were predominantly localized in SP and were less frequent in CP (Fig. 3a, b,c). For the entire provisor sulci stage (12–18gw), the percentage of the NeuN labeling of CP and SP between Cas and Pos was the same (Fig. 4a, b). The ratios of the NeuN labeling CP Cas/CP Pos and SP Cas/SP Pos were approximately equal to one (Fig. 4d, e).

At 19–20 weeks, Pos had smoothed out, while Cas persisted. A notably wide SP under the smoothed Pos was revealed (Fig. 3d). NeuN-immunoreactive cells in the Cas were concentrated mainly in layer VI, whereas in the upper layers, such cells were almost entirely absent (Fig. 3d, e). In the area of the smoothed Pos, the distribution of NeuN-immunoreactive cells was more uniform, and the ratios of NeuN-labeling CP Cas/CP Pos were less than one at these stages (Fig. 4d). The diagram shows the predominance of NeuN labeling in the CP of smoothed Pos (Fig. 4a).

At 20–22 gw, both sulci were detected. The newly emerged Pos was adult-type, the fold was formed by MZ, CP, SP, and IZ and did not involve SVZ and VZ. The distribution of NeuN immunoreactive cells was similar to the previous stages (Fig. 4a). In Pos, NeuN-immunopositive cells were distributed throughout the CP, in Cas – only in the deeper layers of the CP (Fig. 3e, f).

At 23–24 gw, the Cas was smoothed out. A wide SP underlying the Cas was revealed both on Nissl preparations staining and NeuN-labeled sections (Fig. 3g). At this stage, NeuN-immunoreactive cells were found only in the CP and SP. In the Pos area, NeuN- immunoreactivity was significantly lower compared to Cas area (Fig. 4a, b). Statistical differences were found for the calcarine sulcus smoothing stage as well as for middle fetal period (Fig. 4b, c). In the area of the future Cas, there was a predominant localization of NeuN-immunoreactive cells in layers IV, V, and VI. NeuN immunoreactivity clearly defined the boundaries of the primary visual field (Fig. 3g, i,h), which were not yet visible on Nissl preparations. In contrast to previous stages, at 23–24 gw, significant differences in the ratios of NeuN immunoreactivity in Cas and Pos regions were detected for both SP and CP (Fig. 4d, e; Supplement, Tables 4 and 5).

At 25–27 gw, two sulci were distinguished. Signs of cytoarchitectonic differentiation were evident throughout the investigated area by Nissl staining. Two rows of cells in MZ were detected in the Pos region and in areas located dorsal and rostral to this sulcus, defining the boundaries of the secondary visual field. After the 26th gw, the clear NeuN immunoreactivity of layer IV of the primary visual field disappeared; the deeper layers of the CP were labeled predominantly (Fig. 4f). However, stronger NeuN immunoreactivity in the Cas than in the Pos persisted, the ratio CP Cas/CP Pos was greater than one (Fig. 4d).

**Fig. 5:**
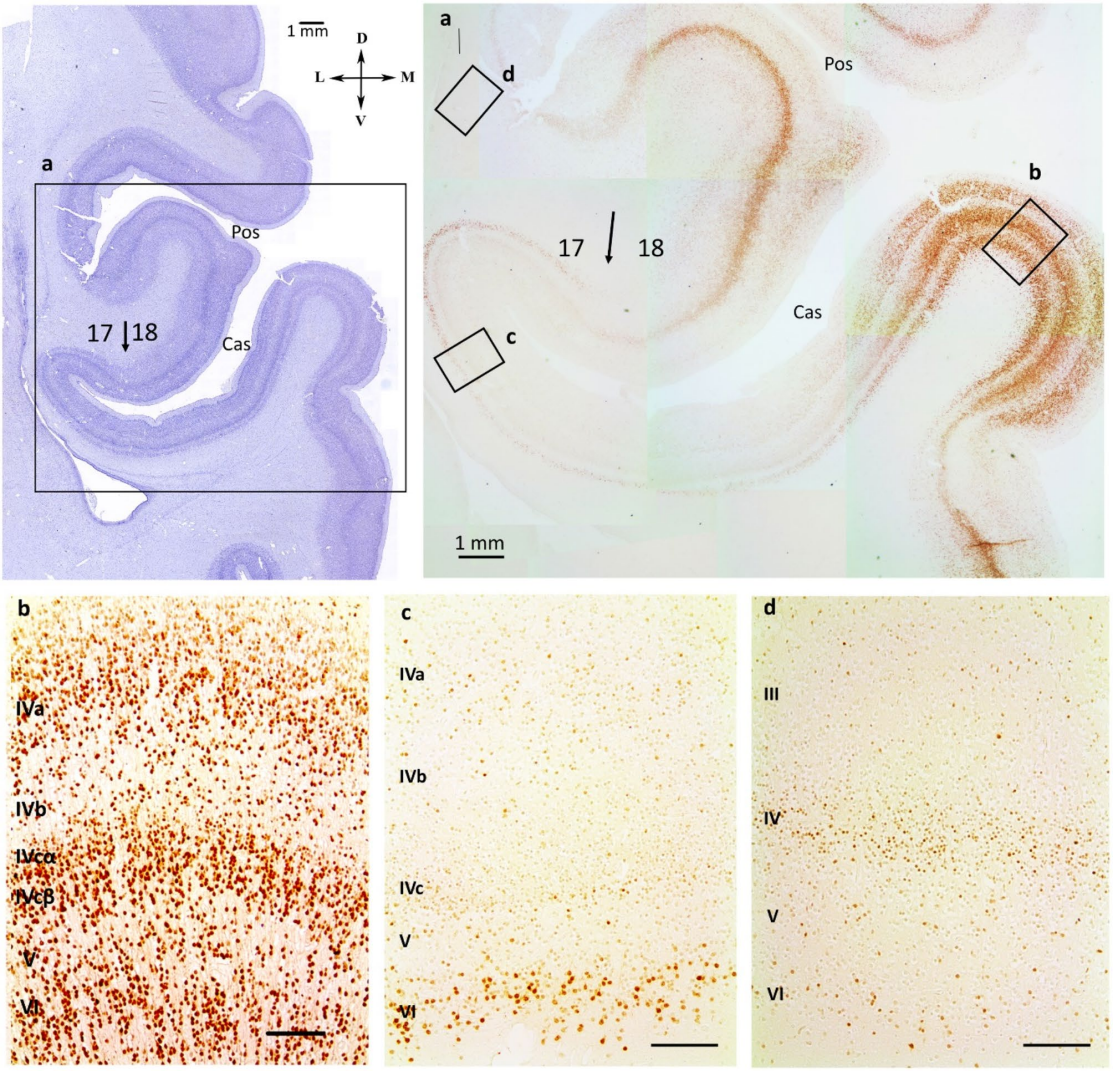
Nissl staining section, the medial part of the occipital lobe, preterm 32 gw + 5 weeks. (**a**) – NeuN-immunohistochemical staining, coronal sections, (**b**) – cortical plate in the bank of the calcarine sulcus (Cas), (**c**) – cortical plate in the fundus of the calcarine sulcus (Cas), (**d**) – cortical plate in the fundus parietooccipital sulcus (Pos). Right hemisphere. Coronal section. Scale bar: 100 μm

At 28–40 gw, there were two sulci. Clear differences in the stratification of CP were visible on Nissl preparations. This stratification corresponds to field 17 in the Cas area, and to field 18 in the Pos area. NeuN immunoreactivity predominated in the banks of the sulci and in the lingual gyrus (*gyrus lingualis*, *gyrus occipitotemporalis medialis)* (Fig. 5a). In the gyrus, labeled cells were presented in all CP layers (Fig. 5b). Inside the sulci, labeling gradually weakened. Near the Cas fundus, NeuN-immunopositive cells were preserved in layer VI. Inside the Pos, immunoreactivity was lower (Figs. 4f and 5c and d). The NeuN immunoreactivity of SP decreased in the late fetal period in both sulci.

Significant differences of the NeuN-immunoreactivity coefficient in the CP of Cas and Pos were found for middle and late fetal periods (Fig. 4c).

Thus, NeuN-immunoreactivity was observed in neuroblasts as early as 12 gw in SVZ, IZ, CP and MZ. At 23–24 gw, in the smoothing Cas area, there was a predominant localization of NeuN-immunoreactive cells in layers IV, V, and VI. NeuN immunoreactivity clearly defined the boundaries of the primary visual field. At the late fetal period, cortical plate of gyri and sulci banks showed higher NeuN-labeling than inside the sulcus in the same cytoarchitectonic field.

### Reelin distribution

**Fig. 6:**
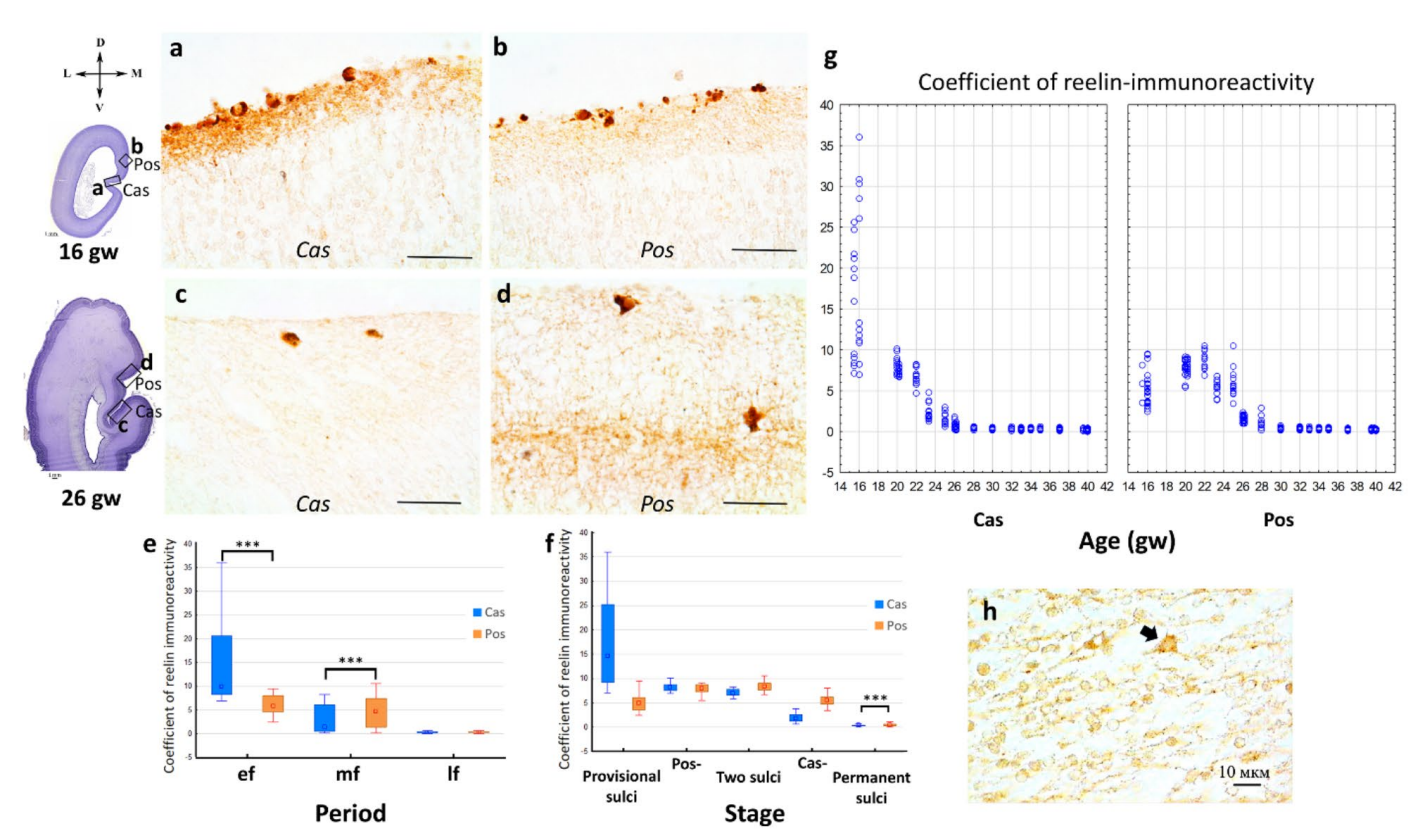
Reelin-positive layer I of the fetal brain: (**a**) – in the calcarine sulcus at 16 gw (Cas); (**b**) – in the parietooccipital sulcus at 16 gw (Pos), right hemisphere, coronal section; (**c**) – in the calcarine sulcus at 26 gw (Cas); (**d**) – in the parietooccipital sulcus at 26 gw (Pos), right hemisphere, coronal section. Dark-stained Cajal–Retzius cells were revealed. Scale bar: 50 μm. (**e**) – Reelin-immunoreactivity coefficient in early fetal (ef), middle fetal (mf) and late fetal (lf) periods. (**f**) – Reelin-immunoreactivity coefficient at different stages: provisional sulci; the parietooccipital sulcus smoothing stage (Pos-); two sulci, when both sulci exist; the calcarine sulcus smoothing stage (Cas-); permanent sulci. (**g**) – Reelin-immunoreactivity coefficient of the layer I at various gestational ages for calcarine sulcus and parietooccipital sulcus. Significant differences between sulci are marked by square brackets and asterisks (***p < 0.0001). (**h**) – Reelin-immunopositive cells in the deep cortical layers, 20 gw. Scale bar: 10 μm

In the Cas and Pos areas, reelin-immunoreactive Cajal–Retzius cells were observed, starting from the 12th gw. The matrix of the marginal zone (I cortical layer) surrounding the Cajal–Retzius cells also exhibited reelin immunoreactivity (Fig. 6a, b). However, as brain development progressed, the immunoreactivity significantly decreased (Fig. 6g; Supplement, Table 2). In the early fetal period, the coefficient of reelin immunoreactivity was higher in the MZ of the Cas than in Pos (Fig. 6e). An opposite distribution of reelin immunoreactivity was revealed in the middle fetal period; the coefficient of reelin immunoreactivity in the MZ of Pos was higher than in Cas (Fig. 6c, d,e). In the late fetal period (28–40 gw), no significant differences were found. At the stages of sulci appearance and smoothing no significant differences were found except for the permanent sulci stage (Fig. 6f).

Between 20 gw and 26 gw, reelin-immunopositive cells were identified in the deep cortical layers (Fig. 6h). These cells did not resemble migrating interneurons, as their localization remained relatively constant. They were usually found within the same level, likely representing future layer IV of the Cas region.

In general, reelin-immunoreactivity of the marginal zone in Cas was increased in the early fetal period and decreased in the middle fetal period. Reelin-immunoreactivity of the marginal zone in Pos was increased later, reaching a peak only in the middle fetal period.

### Immunohistochemical analysis of Ca^2+^-binding protein S-100

**Fig. 7:**
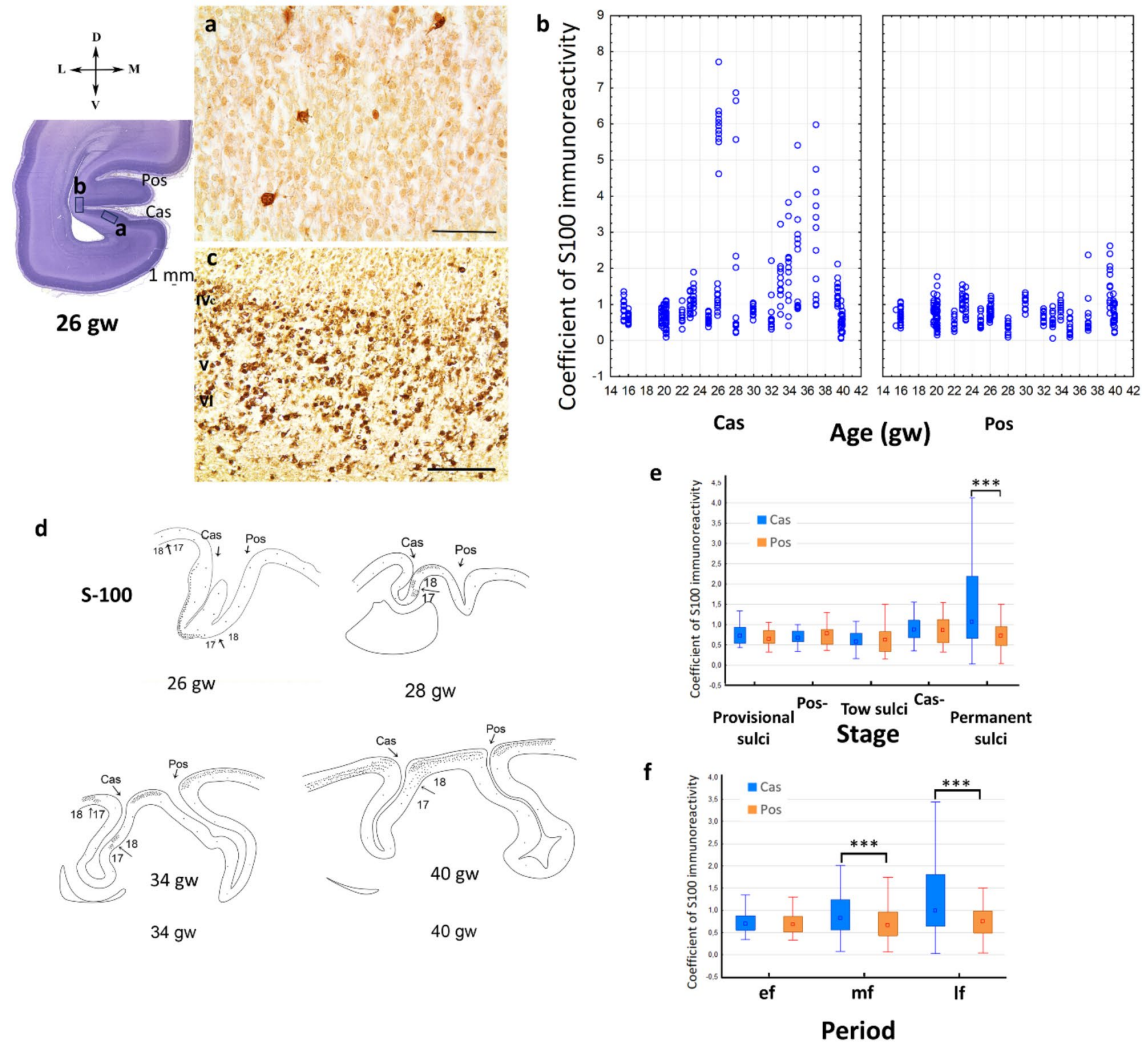
S-100-immunoreactivity in cells in the cortical plate at 26 gw, right hemisphere, coronal section: (**a**) –cells in the cortical plate of the calcarine sulcus (Cas) bank, scale bar: 50 μm; (**b**) – S-100-immunoreactivity coefficient of the layer I at various gestational ages for calcarine sulcus and parietooccipital sulcus. (**c**) - S-100-immunoreactivity in neuroblasts in the fundus of the Cas in the deep cortical layers IVc, V, and VI, scale bar: 100 μm. (**d**) – Scheme of the distribution of S-100-immunoreactive cells in the cortical plate at different stages of fetal development. At 26 gw, S-100-immunoreactive cells are located in deep layers of neuroblasts of the cortical plate in the center of the primary visual field in the calcarine fundus. At 28, 34, and 40 gw, S-100-immunoreactive cells are detected in the gyri, but not in sulci or the border of cytoarchitectonic fields. (**e**) – S-100-immunoreactivity coefficient at different stages: provisional sulci; the parietooccipital sulcus smothing stage (Pos-); two sulci, when both sulci exist; the calcarine sulcus smoothing stage (Cas-); permanent sulci. (**f**) – S-100-immunoreactivity coefficient in early fetal (ef), middle fetal (mf) and late fetal (lf) periods. Significant differences between sulci are marked by square brackets and asterisks (*** *p* < 0.001)

Single S-100-immunopositive cells were diffusely scattered throughout the hemisphere wall of the Cas and Pos from the 12th up to the 26th gw (Fig. 7a). We did not find differences in the distribution of these cells for CP Cas and CP Pos before 26 gw (Fig. 7b, e; Supplement, Table 3). From 26 gw onwards, specific S-100 labeling of cortical neuroblasts was detected in some fetuses (Future 7c).

The first appearance of abundant S-100-immunoreactive neuroblasts was observed at 26gw in the deep cortical layers (IVc, V, and VI), which was prominently marked in the fundus of the Cas (Fig. 7c, d). This immunoreactivity was not evident throughout field 17 and was restricted to the area inside the Cas.

At 28 gw, S-100-immunoreactive neuroblasts were found in layers II and III (field 18) in the upper bank of the Cas and cuneus (*cuneus*) and on the border of fields 17 and 18 (Fig. 7d). Inside the Cas (field 17), S-100-immunoreactive cells were barely presented. It should be emphasized that S-100-positive neuroblasts were detected not throughout the entirety of field 18, but only in the cuneus. There was no S-100 immunoreactivity observed in the opposite lingual gyrus (Fig. 7d).

Between weeks 30–33, this specific S-100-immunoreactive cell distribution was no longer evident. Instead, diffusely localized cells were detected in the hemispheric wall. Starting from 30 gw, S-100-immunoreactive cells demonstrated cytomorphological features of the mature astrocytes.

**Fig. 8:**
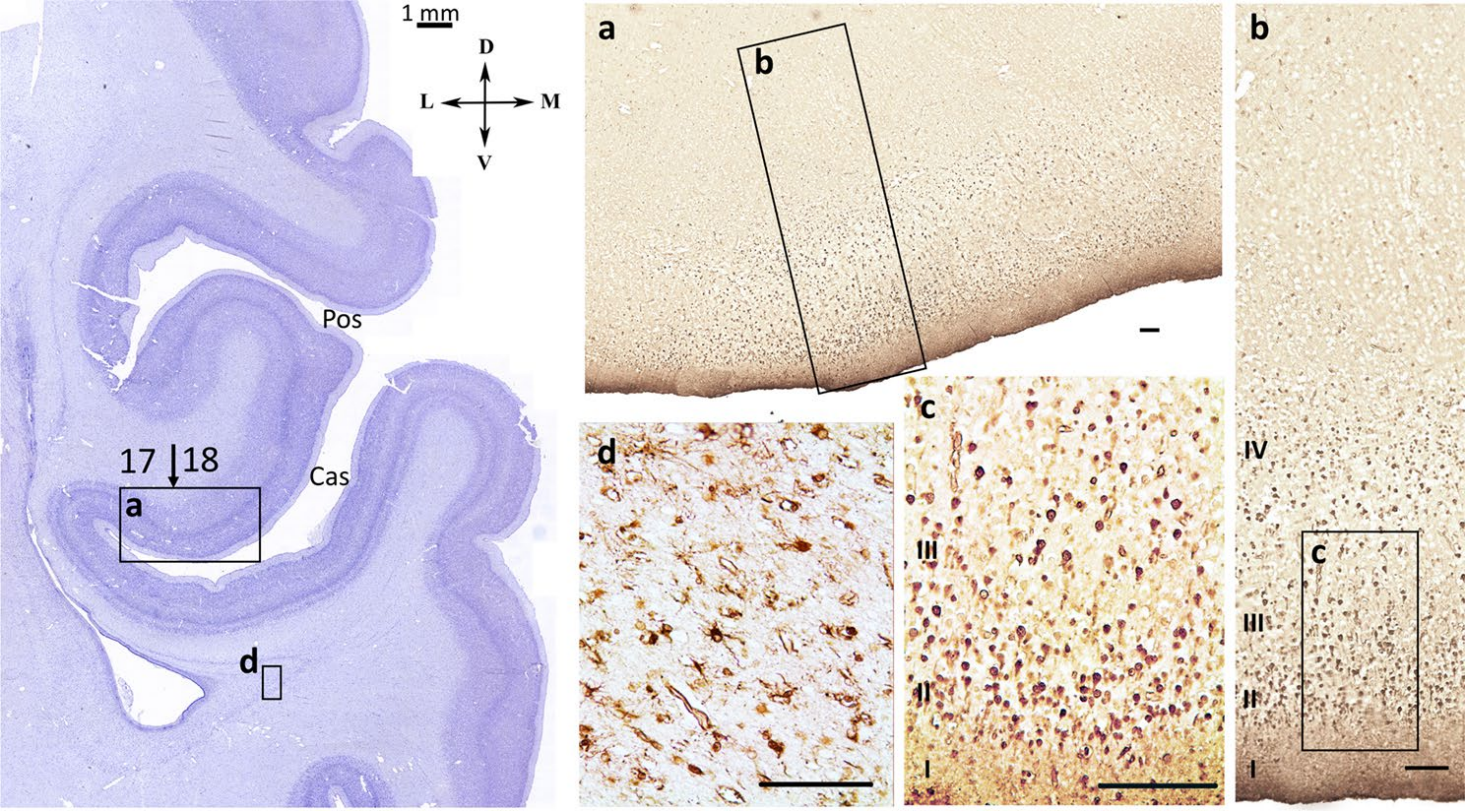
Nissl staining section, the medial part of the occipital lobe, right hemisphere, coronal section, preterm 32 gw + 5 weeks. (**a**, **b**, **c**) – S-100-immunohistochemical staining. Cortical area at the cytoarchitectonic boundary, S-100-immunoreactive neuroblasts, (**d**) – intermediate zone, astrocytes. Scale bar: 100 μm

The second appearance of neocortical S-100-positive neuroblasts was observed from 34 up to 40 weeks. S-100-immunoreactive neuroblasts were predominantly found at the periphery of field 17. Areas of field 18, adjacent to field 17, also possessed S-100 immunoreactivity predominantly at 34–35 gw, although this tendency decreased by week 40 (Figs. 7d and 8a, b and c).

Significant differences in the coefficient of S-100-immunoreactivity between Cas and Pos were observed during the middle and the late fetal period (Fig. 7f) or at the permanent sulci stage (Fig. 7e).

At 26 gw and later in development, S-100-immunoreactive cells with astrocyte cytomorphology were observed in the CP, IZ, and SP (Fig. 8d).

Thus, specific neuronal S-100 labeling was observed in the cortical plate of the Cas fundus at 26 gw. The second appearance of neuronal S-100 labeling was observed from 34 up to 40 weeks at the boundary of fields 17/18 and in the gyrus.

### β-III-tubulin distribution in the visual cortex

**Fig. 9:**
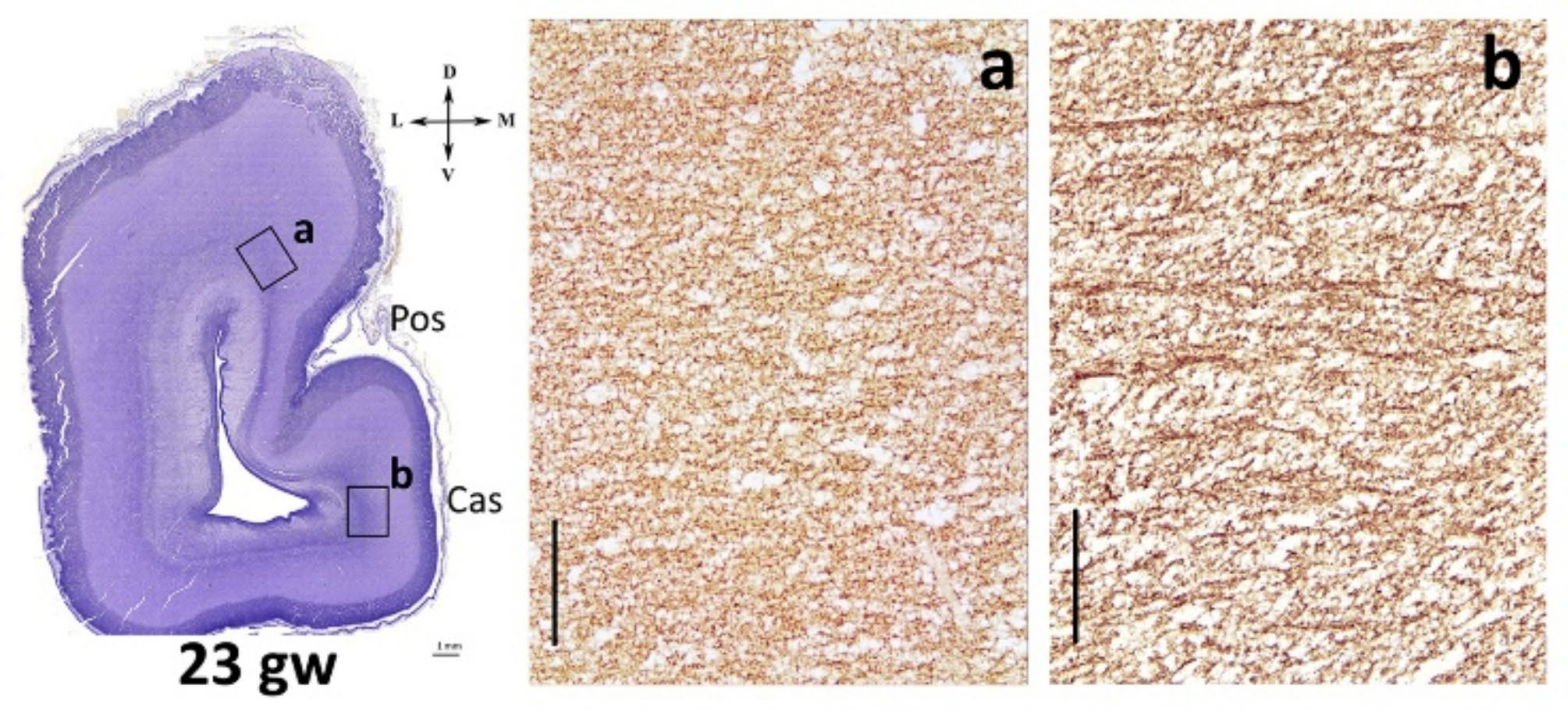
β-III-tubulin-immunoreactive fibers in the intermediate zone (**a**) – dorsomedial cortex, (**b**) – radial β-III-tubulin-immunoreactive bundles of fibers are clearly visible under the smooth cortex of calcarine sulcus at 23 gw. Right hemisphere, coronal section. Scale bar: 100 μm

From the 16 gw onwards, β-III-tubulin immunoreactivity was observed in cells and fibers throughout all transient zones of the hemispheric wall. β-III tubulin-immunoreactivity was also present in the rich nerve fiber network of the IZ and MZ. A dense neural network of β-III-tubulin immunoreactive fibers was detected in the IZ. In the Cas area, beta-III tubulin fibers of the IZ were mainly directed tangentially. This pattern of β-III-tubulin immunoreactivity persisted up to the stage when the Cas smooths out – about 23–24 gw. In the Cas smoothing stage, the pattern of β-III-immunoreactive fibers dramatically changed. Radial β-III-tubulin immunoreactive bundles of fibers were clearly visible beneath the cortical plate of the smoothed Cas (Fig. 9). The IZ of the neighboring regions did not show such obvious radially directed bundles, although the radial orientation of the fibers was more prominent here at previous stages.

In general, the strong radially directed bundles of β-III-tubulin immunoreactive fibers were found in the intermediate zone under the future field 17 at the calcarine sulcus smoothing stage.

## Discussion

### Formation of Cas and Pos during the development of the visual cortex

As already noted in the introduction, studies conducted on rodent brains cannot provide insights into the formation of sulci in the human brain. Unfortunately, there is limited research on the development of the primary and secondary Cas and Pos and there is no consensus regarding their timing in human brain development. According to the book *Pediatric Autopsie* [42] the formation of both Cas and Pos occurs at 16 gw, with reference to an earlier study by Gilles, Leviton, and Pooling, *The Developing Human Brain – Growth and Epidemiological Neuropathology*. However, these sulci are consistently detectable on ultrasound only from 24 to 25 weeks. Recent articles [45, 46] provide data on the possibility of detecting these sulci from 18.5 weeks, but not in all cases and at 20 gw according to Habas et al. [47]. Intrauterine MRI can also reveal the Cas and Pos from 16 weeks [48]. This variation in the occurrence time of Cas and Pos may be a consequence of their temporary smoothing. But only a few works note the fact of temporary smoothing of these sulci during development [28, 29]. Ultrasonic data indirectly point to smoothing, where a decrease in the length of Cas and Pos at similar time intervals is noted [49]. Nevertheless, non-invasive methods have limitations due to their relatively low resolution [29]. In histological preparations in the Atlas of Bayer and Altman [50], Cas had already been observed as early as 11 gw in a fetus with a crown-rump length of 60 mm. In the present study, fetuses with the same crown-rump length showed the presence of both sulci. Moreover, during the period of 13–17 gw, both sulci were found in all cases. It should be noted that these primary sulci represent folds in the entire hemisphere wall, affecting all transient zones, including the ventricular one. Therefore, many studies prefer to refer to such sulci as fissures. Previous results [ [30] have already shown the smoothing of certain primary sulci during prenatal development, likely due to the rapid increase in the size of the hemispheric walls during corticogenesis, followed by the formation of secondary sulci in their constant place. In this paper, the temporary disappearance of the Pos at 17–20 gw (5th lunar month) and Cas at 21–24 gw (6th lunar month) is statistically confirmed.

To support our hypothesis, evidence of sulci disappearance can be observed in the brain sections in the Bayer and Altman printed atlas [50–52] and the online Allen brain atlas [53]. The atlas of Bayer and Altman is a printed edition featuring a series of sections from fetal brains at different developmental stages from the Yakovlev collection of the National Museum of Health and Medicine in the Armed Forces Institute of Pathology, Washington, D.C. Unfortunately, due to volume limitations, not all weeks of development are covered, and the number of sections presented is limited. In the “Developing human brain” from Allen Brain Project sections, there are currently two complete series of fetal brains at 15 and 21 weeks post-fertilization. In both atlases, both Cas and Pos, which are fissures extending through the entire hemisphere wall, are clearly detectable in fetuses up to 17 gw. Unfortunately, the period from 18 to 23 gw is poorly presented, with only 2 samples for this period in the atlas by Bayer and Altman. In one of the presented cases, only the Cas was noted, which aligns with our data on the disappearance (smoothing) of the Pos in the 5th lunar month of development. At 23 gw, both atlases indicate only one Cas which somewhat contradicts our data. However, upon a careful examination of the sections presented in the atlases, we believe that an error may have occurred in their captions as the Pos is actually visible in these sections, and the Cas appears to have temporarily disappeared, as in the cases we studied. This interpretation is supported by the relative positions of the marked sulci in the sections. Additionally, individual variations and interhemispheric asymmetries in brain development could contribute to differences in timing and spatial characteristics of the sulcus appearance [31, 47, 54]. As already noted, sulcus smoothing – is a visible absence of sulcus on its greater length at macromorphological level in comparison with the previous studied stages. According to our observations, the middle part of the calcarine sulcus is smoothed most often. A few millimeters of the anterior and posterior parts of the sulcus may be preserved, as we seen in some studied cases (Supplemental Fig. 2c, d). The presence of residual anterior and posterior parts of the calcarine sulus in some fetuses indirectly indicates that some force raises the bottom of the sulcus. Probably, not always and not in all cases the elevation of the bottom of the sulcus occurs before complete smoothing. We will continue observations of this interesting phenomenon. There is a need for more open-access atlases of the developing human brain with the involvement of a large number of specialists directly engaged in the study of corticogenesis and the formation of sulci and gyri in humans.

### NeuN-immunoreactivity in the development of visual cortex

NeuN is a neuronal nuclear histone protein expressed in cells of both the central and peripheral nervous systems of vertebrae, except for Purkinje cells of the cerebellum, photoreceptor cells of the retina, and mitral cells of the olfactory bulb [44, 55]. Furthermore, neurons in different physiological states can be distinguished by differences in the NeuN/Rbfox3 expression [33]. Similar events were observed during the morphogenesis of Cas and Pos.

Preliminary data was released earlier [56]. According to our new data, NeuN immunoreactivity is detected as early as at the 12th gw. The small folds at the site of future Cas and Pos are already visible at the 12th gw. The first cells migrating to the CP express NeuN. Then we observed the loss of NeuN immunoreactivity in different parts of the neocortex at different time points. In the early fetal period, NeuN immunoreactivity is detected mainly in the SVZ, SP, and deep neurons of the CP and MZ. Earlier studies describe Cajal-Retzius cells as NeuN-immunonegative during the fetal period [57]. Our study reveals NeuN- immunoreactivity of Cajal-Retzius cells in the early fetal period from 12 to 20 gw. In the middle and late fetal periods, NeuN immunoreactivity is typical for the SP and CP. CP in the sulci demonstrates complex appearance and disappearance of NeuN-immunoreactivity. There is a tendency for loss of NeuN immunoreactivity in both the provisor and permanent sulci. The gyri and sulci banks show higher labeling at the late fetal period, which may indicate an earlier maturation of CP in the region, but we will continue to investigate this finding further.

### Heterochronicity of reelin-dependent migration in the visual cortex

Reelin is one of the key molecules in neuroblast migration. Reelin begins to be produced by the Cajal-Retzius cells at the preplate stage (later the marginal zone). Reelin is released into the surrounding matrix and serves as both an attractant and stop-signal for the first neurons migrating from the ventricular zone [34, 35]. The initial neurons in the cortical lamina form the lowest layer VI, starting at 7–8 gw in humans. Subsequent neuroblasts migrating past already established neurons reach the marginal zone and halt their migration. This leads to the development of an inside-out layer forming a 6-layer cortical structure with younger neurons in the outermost region of the brain wall and older neurons inside [58]. Reelin controls the migration and positioning of neuroblasts during cortex formation in the human brain [34, 59, 60]. It is known that reelin is synthesized in the cerebral neocortex and hippocampus by Cajal-Retzius cells during development. It is released by cells into the extracellular space, serving as a reference point for radial glial cells and migrating neuroblasts [60, 61].

Reelin-immunoreactivity is observed in Cajal-Retzius cells of the neocortical anlage in the earliest terms— from the Carnegy stage 16 (about 5 gw) [62]. Reelin immunoreactivity is also detected in the matrix of layer I, surrounding Cajal-Retzius cells. Generally, the earlier formation of the Cas cortex, which contains the future primary visual field 17, is indicated by the distribution pattern of reelin. The intensity of labeling increases earlier in the Cas. Its maximum level is reached at the 16th week of development, and by the 26th week, the reelin labeling in the marginal zone of the Cas reaches the level observed in newborns. In layer I of the Pos, the decrease in the intensity of labeling is delayed, reaching the level observed in newborns only at the 30th week. Thus, the Cas area containing the primary visual field 17 develops more intensively than the area of the Pos, which contains the common anlage of fields 18 and 19.

From 20 to 26 gw, reelin-immunopositive cells are identified in the lower cortical layers. These cells do not resemble migrating interneurons, as their localization remains relatively constant. They are consistently found at the same level, presumably corresponding to the future layer IV. According to the literature, there is a transition from layer-by-layer to diffuse expression of reelin in the postnatal period. In the adult brain, the protein is synthesized by cortical GABAergic interneurons [63], which express calretinin and calbindin, such as Martinotti cells [64, 65]. Thus, the diffuse expression of reelin described in adult rats and primates [65] is also found in human fetuses from the 20th to the 26th week of development.

### The localization of S-100 in neuroblasts of the visual cortex

The heterogeneous occurrence of S-100 protein in different brain structures of human fetuses has been described as appearing earlier in the phylogenetically older structures. In the frontal lobe S-100 was observed at 30 gw according to Zuckerman et al. [66] in the neocortex and hippocampus was observed at 9,5 postconceptional weeks according to Holst et al. [67]. Calcium-binding protein S-100 may act as a growth-differentiating factor for neurons and glia [36] However, there are immunohistochemical data on the distribution of calcium-binding proteins calbindin and parvalbumin in the visual cortex [68], while the detailed expression of S-100 in different fields of the human fetal visual cortex remains unexplored.

In our study, S-100 immunoreactivity in some neocortical neuroblasts is detected in analyzed fetal brain specimens from 26 up to 40 weeks. We assume that this occurrence of S-100 protein in neuroblasts of fetuses during prenatal development from 26 up to 40 weeks is associated with cell differentiation in the prenatal period, although the origin of these cells is a subject of discussion. S-100 is primarily detected in human and mammalian glial cells [69, 70]. There are various isoforms of the S-100 protein. S-100b is detected predominantly in cortical astrocytes [71]. S-100a0, S-100a1, and S-100b can be found in some human and mammalian neuronal populations [37, 72, 73]. Some studies have observed S-100 in neurons of the mammalian brain: rat [74], rabbit, and hamster [75, 76] using polyclonal antibodies. In this study, polyclonal antibodies to S-100 were used, allowing the visualization of not only glial cells but also the temporary neuronal expression of this protein in the human fetal brain.

Based on the pattern of S-100 distribution, an earlier formation of the Cas is observed. The age-depending centrifugal S-100 distribution is revealed: first, S-100 labeling appears in the center of the primary visual field, then – on the periphery of the field, and then in the nearest sites of the secondary field, and so on. Labeling first emerges in the deeper layers (V, VI), and then in the upper layers of the CP.

S-100 is characterized by an increase in the number of labeled cells at the border of cytoarchitectonic fields. This could possibly be the way in which temporal labeling of the visual area subfields may occur [77].

### Heterochrony of visual cortex differentiation, induced by thalamic afferents

According to our data, NeuN immunoreactivity at 23–24 gw makes it possible to visualize the boundaries of the future primary visual field. NeuN immunoreactivity is predominantly detected in layers IV, V, VI, and SP. In the secondary visual field, similar NeuN immunoreactivity is not detected at this age. Ca^2+^-binding protein S-100 marks neuroblasts at the center of the primary visual field starting at 26 gw. S-100-immunopositive cells are located in layers VI, V, and strictly in layer IV. Reelin-immunoreactivity of single cells in layer IV is observed only in the center of the primary visual field at 20–26 gw. We cannot yet describe the exact mechanism but presume that these findings are related. Thus, we assume the beginning of differentiation of the primary visual field from the age of 23–24 gw.

This age (23–25 weeks) is characterized by the ingrowth of afferent thalamic fibers in the primary visual cortex [19, 27, 78, 79]. The significant role of thalamic afferents in cytoarchitectonic differentiation control is revealed by a number of authors in humans and animals [18, 21, 22, 80–84]. The accumulation of thalamic afferents in the subplate of human prenatal period begins at 17 gw, in the cortical plate the first detection of afferents is associated with the period of 21–25 gw [19]. In our study at 23 gw we observed β-III-tubulin labeling of radially oriented bundles of fibers under the primary visual field at 23 gw. The presence of β-III-tubulin in nerve processes makes it possible to label not only neuronal bodies but also axons and dendrites [85]. So, at 23–24 gw we detect: smoothed calcarine sulcus, increasing the width of the subplate below it, a comparative increase in NeuN immunoreactivity in the cortical plate and subplate in this area, β-III-tubulin labeling of radially oriented bundles in the intermediate zone. We speculate that these may be the ingrowth of thalamic afferents from the lateral geniculate body into the primary visual field. It is possible that functional differentiation of the primary visual field begins during the growth of thalamic afferents, which is simultaneously accompanied by smoothing of Cas. However, additional studies of this stage of development are required.

Further functional changes in the primary visual field are revealed by temporary expression of S-100 since 26 gw. The similar intensive marking of layer IV of the primary visual field is revealed using calbindin antibodies (Ca^2+^-binding protein of GABA-ergic neurons) in human fetuses at 26 gestational weeks [68]. Immunoreactivity to other Ca^2+^-binding proteins — parvalbumin, but only in layers V and VI, is revealed at this age. Parvalbumin is shown in sublayer IVc starting only from 30 gw [86]. It is interesting that calbindin is usually not observed in sublayer IVc in the cortex of adults [25, 26, 86]. In contrast, parvalbumin is characteristic of sublayer IVc in field 17 of the differentiated CP [26]. According to our study, polyclonal antibodies to S-100 have no layer-specificity but can label maturing neuroblasts or neuroblasts in a specific functional state.

However, in the article by Letinic and Kostovic [26], the synchronism of Ca^2+^-binding proteins in cortical fields 17 and 18 in postnatal human ontogenesis is emphasized. The synchronous development of primary and secondary fields in primates and human brains has been shown via electron microscopic studies [87, 88]. Nevertheless, studies of synaptic density [89, 90], synaptic activity [91], and electrophysiological analysis [92, 93] testify in favor of the heterogeneity of the primary and secondary fields. Earlier maturation of primary sensory areas has been shown in primates using an immunohistochemical method [94]. Therefore, the data obtained requires further research.

This study demonstrates the heterogeneous formation of fields 17 and 18. In general, it has been shown that the primary visual field matures faster than the secondary one. The earlier smoothing of the Pos compared to the Cas does not lead to the earlier formation of the secondary visual field 18 associated with it. These data may indicate that the processes of increasing the width of the hemisphere due to neuro- and gliogenesis and the processes of cytoarchitectonic maturation of the cortex proceed in parallel. The functional differentiation of the primary visual field begins during the period of thalamic afferent ingrowth. This process coincides with the temporal smoothing of the calcarine sulcus, indicating a simultaneous progression of functional specialization and structural modifications. However, these findings require further confirmation.

## Conclusion

This study has shed light on the intricate processes involved in the development of the human fetal brain, particularly concerning the formation of neocortical sulci and the differentiation of visual fields. Through immunohistochemistry, we were able to study and visualize these processes at the microscopic level, contributing to the understanding of neurodevelopment. Describing cytoarchitecture in accordance with immunoreactivity patterns with specific markers provides additional data on the developmental timing of the human fetal brain. The data obtained can be used in clinical practice to more accurately determine the age of human fetuses. However, the data obtained require further verification involving a larger number of fetal brain studies utilizing both histological and noninvasive methods (ultrasound, MRI). The compilation of detailed, accurate, and terminologically unified reference atlases of the human brain will contribute to addressing issues related to the causes and timing of the formation of primary and secondary sulci.

### Limitations

There are several limitations to this study. First, the small sample size is a major limitation. The second limitation of this study is related to the peculiarities of conducting research on human autopsy material, such as the presence of a spectrum of pathologies in clinical history, including those causing death, and postmortem alterations in tissues. To minimize the effects of postmortem changes and exclude pathological changes in the brain, samples for immunohistochemistry were selected after histological evaluation of the tissue. These samples included spontaneous abortion samples, as well as maternal (diabetes) and fetal (sepsis, cardiac malformations, dystrophy of parenchymal organs) pathology samples. Although these pathologies have no evidenced effects on brain development, such effects cannot be absolutely excluded. Other limitations can be found in the text of this article.

## Electronic supplementary material

Below is the link to the electronic supplementary material.

Supplementary Material 1

## Data Availability

The anonymized complete dataset will be provided as a basis for further investigations on the special Human Brain Development Atlas website at https://brainmorphology.science/(accessed on 26 September 2023). Part of the Collection samples is also publicly accessible via the laboratory website at https://brainmicroscopy.com/collection/homo/brain-development/ (accessed on 26 September 2023).
