# Supplementary material for: Heterogeneity in the formation of primary and secondary visual fields during human prenatal development
Source: Biol Res. 2024 Nov 28;57:93. doi: 10.1186/s40659-024-00576-0 (PMC11603890; doi:10.1186/s40659-024-00576-0)
Supplement: Supplementary file 1 — Supplementary Material 1 [file 40659_2024_576_MOESM1_ESM.docx]

**Table 1**. Characteristics of the investigated fetuses.

| Pa | CRL^1^ (mm) | Heigt  (mm) | Weight (g) | Age (gw^2^) | Sex | Cause of death | Fixation | Immunohistochemistry |
| --- | --- | --- | --- | --- | --- | --- | --- | --- |

| 1 | 50 | N/A3 | N/A | 12 | Male | Medical legal abortion | Buffered formalin | Yes |
| --- | --- | --- | --- | --- | --- | --- | --- | --- |
| 2 | 60 | N/A3 | N/A3 | 13 | N/A3 | N/A3 | Acidic formalin | No |
| 3 | 75-78 | N/A3 | N/A3 | 14 | N/A3 | Uteroctomy | Acidic formalin | No |
| 4 | N/A | N/A | N/A | 15 | Female | Medical legal abortion on social grounds | Acidic formalin | No |
| 5 | 110 | N/A | N/A | 15 | Female | Spontaneous abortion | Acidic formalin | No |
| 6 | N/A | N/A | N/A | 16 | Male | Spontaneous abortion | Acidic formalin | No |
| 7 | N/A | N/A | N/A | 16 | N/A | N/A | Acidic formalin | No |
| 8 | 115 | N/A | N/A | 16 | Male | Spontaneous abortion | Buffered formalin | Yes |
| 9 | N/A | N/A | N/A | 16 | Female | Therapeutic abortion  (tubal pregnancy) | Buffered formalin | Yes |
| 10 | N/A | 170 | 90 | 16 | Female | Therapeutic abortion  (mother severe diabetes-I) | Buffered formalin | Yes |
| 11 | 130 | N/A | N/A | 17 | Female | N/A | Acidic formalin | No |
| 12 | 132 | N/A | N/A | 17 | Female | N/A | Acidic formalin | No |
| 13 | 135 | N/A | N/A | 17 | Male | N/A | Acidic formalin | No |
| 14 | 148 | N/A | N/A | 18 | Female | N/A | Acidic formalin | No |
| 15 | N/A | N/A | N/A | 18 | Female | Spontaneous abortion | Acidic formalin | No |
| 16 | N/A | N/A | 230 | 20 | Female | Therapeutic abortion (mother’s medical reason) | Acidic formalin | No |
| 17 | N/A | N/A | N/A | 19-20 | Male | Spontaneous abortion | Buffered formalin | Yes |
| 18 | 168 | N/A | 234 | 20 | Male | Therapeutic abortion  (mother severe diabetes-I) | Buffered formalin | Yes |
| 19 | 160 | N/A | N/A | 20 | Male | Spontaneous abortion  (one of dichorionic-diamniotic twins) | Buffered formalin | Yes |
| 20 | 145 | N/A | N/A | 19 | Female | N/A | Acidic formalin | No |
| 21 | 150 | N/A | N/A | 19 | Male | An accident, the fetus of a deceased pregnant woman | Acidic formalin | No |
| 22 | 173 | 270 | 331 | 21 | Male | Spontaneous abortion | Buffered formalin | Yes |
| 23 | N/A | N/A | 390 | 21 | Female | Therapeutic abortion (mother’s medical reason) | Acidic formalin | No |
| 24 | N/A | N/A | 410 | 23 | Female | N/A | Acidic formalin | No |
| 25 | N/A | N/A | N/A | 21 | Female | N/A | Acidic formalin | No |
| 26 | N/A | N/A | N/A | 21-22 | Male | Therapeutic abortion (mother’s medical reason) | Acidic formalin | No |
| 27 | N/A | N/A | N/A | 21-22 | Male | Therapeutic abortion (mother’s medical reason) | Acidic formalin | No |
| 28 | N/A | N/A | 600 | 22 | Female | N/A | Acidic formalin | No |
| 29 | N/A | N/A | 470 | 22 | Male | Medical legal abortion on social grounds | Acidic formalin | No |
| 30 | N/A | 290 | 490 | 22-23 | Female | Preterm  (intrauterine pneumonia, dystrophy of parenchymal organs) | Buffered formalin | Yes |
| 31 | N/A | N/A | N/A | 23 | Male | N/A | Buffered formalin | Yes |
| 32 | 235 | 310 | 622 | 23 | Female | Spontaneous abortion | Buffered formalin | Yes |
| 33 | N/A | N/A | N/A | 24 | Male | Spontaneous abortion | Buffered formalin | Yes |
| 34 | N/A | 340 | 870 | 26 | Female | Intranatal fetal death (right-sided hypoplastic heart) | Buffered formalin | Yes |
| 35 | N/A | N/A | N/A | 25 | Male | N/A | Acidic formalin | No |
| 36 | N/A | 340 | 880 | 26+2 days | Female | Preterm  (sepsis, respiratory distress syndrome, secondary immune deficiency) | Buffered formalin | Yes |
| 37 | N/A | 310 | 730 | 26 | Female | Intranatal fetal death (mother severe diabetes-I) | Bouin's solution | Yes |
| 38 | 240 | N/A | N/A | 27 | Male | N/A | Acidic formalin | No |
| 39 | N/A | N/A | 530 | 26 | Male | N/A | Acidic formalin | No |
| 40 | N/A | N/A | N/A | 26 | Female | N/A | Acidic formalin | No |
| 41 | N/A | 370 | 1020 | 28 | Female | Preterm  (intraventricular hemorrhage, mother severe diabetes-I) | Bouin's solution | Yes |
| 42 | 285 | N/A | N/A | 32 | Male | N/A | Acidic formalin | No |
| 43 | N/A | 390 | 1410 | 30+17 days | Female | Preterm  (intrauterine sepsis, respiratory distress syndrome, infective toxic shock) | Buffered formalin | No |
| 44 | N/A | 370 | 1250 | 28+14 days | Male | (Preterm  (intrauterine sepsis, respiratory distress syndrome) | Buffered formalin | No |
| 45 | N/A | 390 | 1400 | 30+5 days | Female | Preterm  (intrauterine sepsis, adrenal hyperplasia) | Buffered formalin | Yes |
| 46 | N/A | N/A | n/d | 30-31 | Male | N/A | Buffered formalin | Yes |
| 47 | N/A | N/A | 1500 | 30+10 days | Male | Preterm  (bilateral pneumonia, bilateral pneumothorax congenital heart disease) | Bouin's solution | Yes |
| 48 | N/A | N/A | N/A | 32 | Female | N/A | Acidic formalin | No |
| 49 | N/A | 410 | 1500 | 32+8 days | Male | Preterm  (intrauterine sepsis, bilateral pneumothorax) | Buffered formalin | Yes |
| 50 | n/d | 450 | 1640 | 34+9 days | Male | Preterm  (intrauterine sepsis, respiratory distress syndrome) | Buffered formalin | Yes |
| 51 | N/A | N/A | 1420 | 32+5 weeks | Male | Preterm  (intrauterine infection, pneumonia) | Bouin's solution | Yes |
| 52 | N/A | 410 | 3670 | 34-35 | Male | Intrauterine fetal death | Bouin's solution | Yes |
| 53 | N/A | N/A | N/A | 35+5 weeks | Female | Preterm (intrauterine infection, pneumonia) | Bouin's solution | Yes |
| 54 | N/A | 490 | 3080 | 37+6 days | Male | Intrauterine infection, multiple malformations | Buffered formalin | No |
| 55 | N/A | N/A | 2950 | 40 | Male | Intrauterine infection, pneumonia | Bouin's solution | Yes |
| 56 | N/A | 460 | 2900 | 40 | Female | Multiple malformations | Buffered formalin | Yes |

^1^CRL – crown-rump length, ^2^gw – gestational weeks, ^3^N/A- data is not available.

**Table 2**. Reelin-immunoreactivity coefficient in the marginal zone of the calcarine (Cas) and parietooccipital sulci (Pos).

| Period | Age, gw | Cas (n=12) | Pos (n=12) |
| --- | --- | --- | --- |
| Ef | 16 | \| 17.4055*(8.6985-8.6985) \| \| --- \| | \| 4.896 (4.0345-6.105) \| \| --- \| |
| ef | 16 | \| 12.876 (11.0015-29.418) \| \| --- \| | \| 5.08 (3.2845-6.380) \| \| --- \| |
| ef | 20 | \| 8.225 (7.5015-8.7980) \| \| --- \| | \| 7.9275 (7.311-8.7415) \| \| --- \| |
| ef | 20 | \| 7.1725 (6.815-7.813) \| \| --- \| | \| 7.7415 (7.155-8.631) \| \| --- \| |
| mf | 22 | \| 6.6175 (6.1115-7.5195) \| \| --- \| | \| 8.967 (7.9485-9.5705) \| \| --- \| |
| mf | 23 | \| 2.021 (1.708-3.068) \| \| --- \| | \| 5.3325 (4.6755-5.866) \| \| --- \| |
| mf | 26 | \| 1.246 (1.072-2.0825) \| \| --- \| | \| 5.5345 (4.8615-6.7385) \| \| --- \| |
| mf | 26 | \| 0.647 (0.4445-1.0135) \| \| --- \| | \| 1.5 (1.18-1.941) \| \| --- \| |
| mf | 26 | \| 0.432 (0.3185-0.688) \| \| --- \| | \| 1.438 (1.1445-1.89) \| \| --- \| |
| mf | 28 | \| 0.47 (0.318-0.5295) \| \| --- \| | \| 0.885 (0.6165-1.228) \| \| --- \| |
| lf | 30 | \| 0.2955 (0.2085-0.425) \| \| --- \| | \| 0.449 (0.315-0.5405) \| \| --- \| |
| lf | 35 | \| 0.372 (0.271-0.407) \| \| --- \| | \| 0.337 (0.246-0.383) \| \| --- \| |
| lf | 32 | \| 0.401 (0.3085-0.463) \| \| --- \| | \| 0.33 (0.239-0.4725) \| \| --- \| |
| lf | 37 | \| 0.36 (0.257-0.408) \| \| --- \| | \| 0.3315 (0.2655-0.4345) \| \| --- \| |
| lf | 34 | \| 0.2285 (0.1315-0.379) \| \| --- \| | \| 0.2495 (0.2115-0.322) \| \| --- \| |
| lf | 35 | \| 0.3175 (0.2225-0.4135) \| \| --- \| | \| 0.328 (0.2335-0.41) \| \| --- \| |
| lf | 40 | \| 0.311 (0.1355-0.333) \| \| --- \| | \| 0.279 (0.145-0.3355) \| \| --- \| |
| lf | 40 | \| 0.2335 (0.16-0.329) \| \| --- \| | \| 0.322 (0.292-0.3595) \| \| --- \| |
| lf | 40 | \| 0.3115 (0.177-0.331) \| \| --- \| | \| 0.225 (0.1395-0.2795) \| \| --- \| |

* Values are presented as the median and upper and lower quartile values (Me (q1–q3)) and the number of measurements (n).

**Table 3.** S-100-immunoreactivity coefficient values in the cortical plate of the calcarine (Cas) and parietooccipital sulci (Pos).

| Period | Age, gw | Cas (n=12) | Pos (n=12) |
| --- | --- | --- | --- |
| ef | 16 | \| 0.9315* (0.764-1.0625) \| \| --- \| | \| 0.6465 (0.5478-0.7895) \| \| --- \| |
| ef | 16 | \| 0.6185 (0.492-0.714) \| \| --- \| | \| 0.6435 (0.5698-0.86) \| \| --- \| |
| ef | 20 | \| 0.6785 (0.5055-0.8155) \| \| --- \| | \| 0.794 (0.51-0.97) \| \| --- \| |
| ef | 20 | \| 0.6905 (0.663-0.89) \| \| --- \| | \| 0.6510 (0.4645-0.8065) \| \| --- \| |
| mf | 21 | \| 0.3935 (0.242-0.529) \| \| --- \| | \| 0.3285 (0.2525-0.5365) \| \| --- \| |
| mf | 21 | \| 0.769 (0.681-0.969) \| \| --- \| | \| 1.018 (0.7865-1.2390) \| \| --- \| |
| mf | 22 | \| 0.905 (0.7835-1.104) \| \| --- \| | \| 1.0685 (0.9685-1.2385) \| \| --- \| |
| mf | 23 | \| 0.5865 (0.5155 -0.807) \| \| --- \| | \| 0.505(0.379-0.6505) \| \| --- \| |
| mf | 23 | \| 1.102 (1.004 -1.3905) \| \| --- \| | \| 0.9145 (0.6145-1.2055) \| \| --- \| |
| mf | 26 | \| 0.6255 (0.5055-0.722) \| \| --- \| | \| 0.4065(0.376-0.6030) \| \| --- \| |
| mf | 26 | \| 1.053(0.9075-1.2615) \| \| --- \| | \| 0.6555 (0.597-0.8315) \| \| --- \| |
| mf | 26 | \| 5.8775 (5.6545-6.1855) \| \| --- \| | \| 0.917 (0.771-1.0545) \| \| --- \| |
| mf | 28 | \| 0.533 (0.313-3.9375) \| \| --- \| | \| 0.3675 (0.181-0.4885) \| \| --- \| |
| lf | 30 | \| 0.8195 (0.693-0.967) \| \| --- \| | \| 1.0395 (0.8505-1.1415) \| \| --- \| |
| lf | 33 | \| 1.557 (1.315-1.9255) \| \| --- \| | \| 0.5645 (0.4515-0.674) \| \| --- \| |
| lf | 35 | \| 1.742 (0.873-2.285) \| \| --- \| | \| 0.868 (0.804-0.985) \| \| --- \| |
| lf | 32 | \| 0.5665 (0.389-1.0105) \| \| --- \| | \| 0.674 (0.508-0.817) \| \| --- \| |
| lf | 37 | \| 2.807 (1.1885-3.9135) \| \| --- \| | \| 0.4705 (0.3855-0.6995) \| \| --- \| |
| lf | 34 | \| 2.5825 (0.9475-3.1325) \| \| --- \| | \| 0.2495 (0.1815-0.5225) \| \| --- \| |
| lf | 40 | 1.272 (1.13-1.5088) | 1.4805 (1.0825-19765) |
| lf | 40 | 0.387 (0.3098-0.56993) | 0.8245 (0.5325-0.9865) |
| lf | 40 | 0.567 (0.3923-0.6978) | 0.6565 (0.482-0.69775) |

* Values are presented as the median and upper and lower quartile values (Me (q1–q3)) and the number of measurements (n).

**Table 4**. NeuN-immunoreactivity coefficient in the cortical plate of the calcarine and parietooccipital sulci.

| Period | Stage | Age, gw | Cas (n=12) | Pos (n=12) |
| --- | --- | --- | --- | --- |
| ef | Provisional sulci | 12 | 19.4705^1^ (15.6563-22.504) | 16.843 (13.4685-17.9693) |
| ef | Provisional sulci | 16 | \| 1.8625 (1.6755-2.1825) \| \| --- \| | \| 1.546 (1.3078-1.82205) \| \| --- \| |
| ef | Pos -^2^ | 20 | \| 0.5995 (0.4058-0.879) \| \| --- \| | 8.505 (8.0263-8-8435) |
| mf | Two sulci | 22 | 4.441 (3.4618-5.7243) | 8.4645 (8.0655-8.803) |
| mf | Cas -^3^ | 23 | \| 2.696 (2.2078-3.5783) \| \| --- \| | \| 0.4915 (0.3973-0.5538) \| \| --- \| |
| mf | Cas - | 24 | \| 1.9075 (1.615-2-4825) \|  \| \| --- \| --- \| | \| 0.092 (0.0793-0.109) \| \| --- \| |
| mf | Permanent sulci | 26 | 10.525 (10.1248-10.8783) | 9.622 (9.0728-9.8203) |
| mf | Permanent sulci | 28 | 11.102 (19.5435-11.57558) | 7.2255 (6.4225-7.9778) |
| lf | Permanent sulci | 32 | 6.321 (5.827-6.6778) | 2.9405 (2.8915-3.1583) |
| lf | Permanent sulci | 33 | 5.6035 (4.509-8-484) | 0.4995 (0.3453-2.4463) |
| lf | Permanent sulci | 37 | 1.6525 (1.3983)-2.5565) | 0.6055 (0.4848-1.5333) |
| lf | Permanent sulci | 40 | 1.877 (1.4755-3.0118) | 0.9025 (0.7475-1.1013) |

^1^ Values are presented as the median and upper and lower quartile values (Me (q1–q3)) and the number of measurements (n); ^2^parietooccipital sulcus smoothing stage (Pos-); ^3^ two sulci, when both sulci exist; calcarine sulcus smoothing stage (Cas-).

**Table 5**. NeuN-immunoreactivity coefficient in the subplate of the calcarine and parietooccipital sulci.

| Period | Stage | Age, gw | Cas (n=12) | Pos (n=12) |
| --- | --- | --- | --- | --- |
| ef | Provisional sulci | 12 | - | - |
| ef | Provisional sulci | 16 | 7.0805^1^ (6.6418-7.556) | 6.046 (5.9435-6.284) |
| ef | Pos -^2^ | 20 | 0.273 (0.2395-0.321) | 0.4235 (0.3758-0.806) |
| mf | Two sulci | 22 | 1.0975 (0.9515-1.2655) | 0.97 (0.91-1.0845) |
| mf | Cas -^3^ | 23 | 1.3445 (1.07-2.3483) | 0.24 (0.142-0.2768) |
| mf | Cas - | 24 | 0.329 (0.2798-0.378) | 0.1125 (0.1073-0.1335) |
| mf | Permanent sulci | 26 | 0.898 (0.743-1.0893) | 1.123 (0.9095-1.2933) |
| mf | Permanent sulci | 28 | 0.917 (0.863-1.0415) | 2.146 (2.012-2.2558) |
| lf | Permanent sulci | 32 | 0.4405 (0.3923-0.5503) | 0.722 (0.4878-0.8183) |
| lf | Permanent sulci | 33 | 0.115 (0.10375-0.1293) | 0.096 (0.0785-0.1253) |
| lf | Permanent sulci | 37 | 0.0845 (0.0443–0.1048) | 0.1005 (0.0718-0.123) |
| lf | Permanent sulci | 40 | 0.0815 (0.0618-0.1005) | 0.082 (0.0648-0.097) |

^1^ Values are presented as the median and upper and lower quartile values (Me (q1–q3)) and the number of measurements (n); ^2^parietooccipital sulcus smoothing stage (Pos-); ^3^ two sulci, when both sulci exist; calcarine sulcus smoothing stage (Cas-).


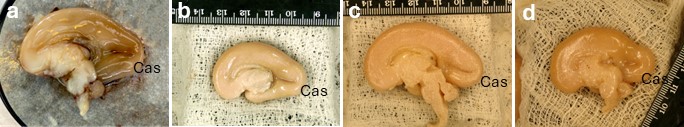


**Figure 1**. Human fetal brains with visible calcarine sulcus and smoothed parieto-occipital sulcus: (a) – 18 gw, right hemisphere; (b) – 19-20 gw, right hemisphere; (c) – 20 gw, right hemisphere; (d) – 18 gw, right hemisphere.


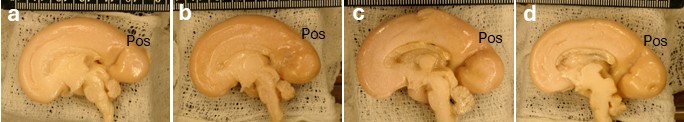


**Figure 2**. Human fetal brains with visible parieto-occipital sulcus and smoothed calcarine sulcus: (a) – 21-22 gw, right hemisphere; (b) – 22 gw, right hemisphere; (c) – 22 gw, right hemisphere; (d) – 23 gw, right hemisphere.
